# An artificial intelligence interpretable tool to predict risk of deep vein thrombosis after endovenous thermal ablation

**DOI:** 10.1016/j.jvsv.2025.102253

**Published:** 2025-04-30

**Authors:** Azadeh Tabari, Yu Ma, Jesus Alfonso, Anthony Gebran, Haytham Kaafarani, Dimitris Bertsimas, Dania Daye

**Affiliations:** a Department of Radiology, Massachusetts General Hospital, Boston, MA; b Harvard Medical School, Boston, MA; c Operations Research Center, Massachusetts Institute of Technology, Cambridge, MA; d Trauma, Emergency Surgery, and Surgical Critical Care Department, Massachusetts General Hospital, Boston, MA

**Keywords:** Artificial intelligence, Chronic venous disease, Endovascular thermal ablation, Radiofrequency ablation, Superficial venous disease, Venous stripping

## Abstract

**Objective:**

Endovenous thermal ablation (EVTA) stands as one of the primary treatments for superficial venous insufficiency. Concern exists about the potential for thromboembolic complications following this procedure. Although rare, those complications can be severe, necessitating early identification of patients prone to increased thrombotic risks. This study aims to leverage artificial intelligence-based algorithms to forecast patients’ likelihood of developing deep vein thrombosis (DVT) within 30 days following EVTA.

**Methods:**

From 2007 to 2017, all patients who underwent EVTA were identified using the American College of Surgeons National Surgical Quality Improvement Program database. We developed and validated four machine learning models using demographics, comorbidities, and laboratory values to predict the risk of postoperative DVT: Classification and Regression Trees (CART), Optimal Classification Trees (OCT), Random Forests, and Extreme Gradient Boosting (XGBoost). The models were trained using all the available variables. SHapley Additive exPlanations analysis was adopted to interpret model outcomes and offer medical insights into feature importance and interactions.

**Results:**

A total of 21,549 patients were included (mean age, 54 ± 14 years; 67% female). In this cohort, 1.59% developed DVT. The XGBoost model had good discriminative power for predicting DVT risk with area under the curve of 0.711 in the hold-out test set for the all-variable model. Stratification of the test set by age, body mass index, preoperative white blood cell count, and platelet count shows that the model performs equally well across these groups.

**Conclusions:**

We developed and validated an interpretable model that enables physicians to predict which patients with superficial venous insufficiency has higher risk of developing DVT within 30 days following EVTA.

**Article Highlights**

• **Type of Research:** Retrospective cohort study utilizing a national surgical outcomes database to develop and validate predictive models for postprocedural complications Retrospective cohort study utilizing a national surgical outcomes database to develop and validate predictive models for postprocedural complications
• **Key Findings:** A gradient-boosted decision tree model (XGBoost) accurately predicted the 30-day risk of deep vein thrombosis after endovenous thermal ablation, with key predictors including body mass index, preoperative platelet count, white blood cell count, and patient age.
• **Take Home Message:** A validated, interpretable risk prediction tool can help clinicians identify patients at increased risk for thrombotic events after endovenous thermal ablation and support more personalized postoperative care strategies.

There has been rapid growth in the use of endovenous thermal ablation (EVTA) of varicose veins.^1^ In 2013, the National Institute for Health and Care Excellence recommended EVTA as the preferred treatment option for symptomatic venous insufficiency.^2^ This treatment approach induces heat-mediated vessel wall injury, resulting in thrombotic and fibrotic occlusion, which raises concerns about the potential risk of venous thromboembolism (VTE).^3^ Although the complications of deep venous thrombosis (DVT) and pulmonary embolism are believed to be infrequent, the Society for Vascular Surgery advises patients to undergo early post-procedural duplex scanning to detect potential thrombotic events.^4^^,^^5^ Notably, the European Society for Vascular Surgery does not make such a recommendation.^6^ The routine use of duplex surveillance has led to the recognition of a new form of localized postoperative DVT, which is termed endovenous heat-induced thrombosis, referring to the extension of thrombus from the ablated superficial vein into the deep vein.^7^ Endovenous heat-induced thrombosis rates reported in the literature vary widely from 0% to 8%, with no consensus on its management.^8^ Given the substantial volume of EVTA procedures performed globally and the potential for severe complications, it is imperative for health care providers to have a clear understanding of the true incidence of VTE complications.^7^^,^^8^ Such insights can aid in individual patient decision-making and facilitate research on VTE prevention strategies.^9^^,^^10^ To address these concerns, we have developed machine learning models to predict the 30-day procedure-related risk of DVT in patients undergoing lower extremity EVTA.^11^ Leveraging the American College of Surgeons National Surgical Quality Improvement Program (ACS-NSQIP)—a comprehensive, multi-hospital database—we constructed and assessed machine learning models for DVT risk prediction. Each model was analyzed to identify key risk factors, and rigorous testing was conducted to ensure the models’ robustness across diverse demographic groups (race, sex, and age), thus enhancing their applicability in various care settings with differing patient populations.

## Methods

### Patient selection

A cohort of 21,549 patients who underwent endovenous thermal ablation from January 1, 2007, to December 31, 2017, across 700 hospitals in the United States was identified using data from the ACS-NSQIP database.^12^ The data were obtained from a national registry, ensuring patient anonymity. The study encompassed procedures performed over a 10-year period. Exclusions from the ACS-NSQIP dataset included minor cases, patients under 18 years old, cases with an American Society of Anesthesiologists (ASA) score of 6 (brain-death organ donors), trauma cases, transplant cases, cases exceeding the NSQIP contract’s specified number, returns to the operating room due to complications of prior procedures, and cases with a prior NSQIP-assessed procedure within 30 days. Data subsets were created using Current Procedural Terminology codes to include patients who underwent EVTA for superficial venous insufficiency.

### Definitions and aim

Patient demographics, laboratory findings, and clinical variables, including lab values and 30-day postoperative risk of DVT, were obtained from the ACS-NSQIP database. The ACS-NSQIP repository also offers a targeted vascular module with pre- and postoperative variables specific to vascular disease and the type of procedure, which was merged with the previously selected cases to provide additional granular details about the procedure performed. A comprehensive table of the 21,549 observations and 34 variables is presented below. The dataset encompasses a wide range of features, including demographic details, clinical indicators, and laboratory results, which are outlined in Table I. This table summarizes the entire cohort, with mean age of patients used for model development being 54.04 (standard deviation [SD], 13.7) years. Our main objective was to identify clinically relevant factors linked to the incidence of DVT within 30 days following EVTA.

**Table I:** Patient population demographics and clinical variables

	Training	Testing	Total	*P*
No. (%)	16,161	5388	21,549	
Demographics and race				
Male	5320 (32.92)	1759 (32.6)	7079 (32.85)	.67
Ethnicity – Hispanic	1512 (9.36)	518 (9.61)	2030 (9.42)	.52
Race				
American Indian or Alaska Native	207 (1.28)	54 (1.0)	261 (1.2)	.07
Asian	560 (3.47)	176 (3.27)	736 (3.4)	.43
Black or African American	627 (3.88)	228 (4.23)	855 (4.0)	.18
White	14,667 (90.76)	4890 (90.76)	19,557 (90.8)	1.00
Native Hawaiian or Pacific Islander	100 (0.62)	40 (0.74)	140 (0.6)	.25
Clinical comorbidities and presenting symptomatology				
Age, years	54.04 (13.81)	54.03 (13.73)	54.04 (13.79)	.98
ASA class				
1 – no disturb	2932 (18.14)	973 (18.06)	3905 (18.12)	.68
2 – mild disturb	9574 (59.24)	3210 (59.58)	12,784 (59.33)	.68
3 – severe disturb	3485 (21.56)	1144 (21.23)	4629 (21.48)	.68
4 – life threat	166 (1.03)	61 (1.13)	227 (1.05)	.68
5 – moribund	4 (0.02)	0 (0.00)	4 (0.02)	.68
Smoker	1924 (11.91)	677 (12.56)	2601 (12.07)	.14
COPD	315 (1.95)	102 (1.89)	417 (1.94)	.77
Ascites	4 (0.02)	1 (0.02)	5 (0.02)	.77
CHF	32 (0.20)	14 (0.26)	46 (0.21)	.31
Hypertension	5522 (34.17)	1816 (33.70)	7338 (34.05)	.47
Renal failure	9 (0.06)	1 (0.02)	10 (0.05)	.25
Bleeding disorder	494 (3.06)	159 (2.95)	653 (3.03)	.65
Transfusion requirement	5 (0.03)	2 (0.04)	7 (0.03)	.80
BMI, kg/m^2^	30.47 (7.66)	30.38 (7.58)	30.45 (7.64)	.44
Preoperative labs				
Sodium	139.57 (2.07)	139.59 (2.10)	139.58 (2.07)	.58
BUN	15.75 (5.75)	15.73 (5.88)	15.75 (5.78)	.78
Creatinine	0.87 (0.50)	0.87 (0.47)	0.87 (0.49)	.75
WBC	6.63 (1.72)	6.64 (1.83)	6.63 (1.75)	.56
Hematocrit	40.58 (3.41)	40.51 (3.46)	40.56 (3.43)	.20
Platelet	239.94 (55.07)	240.50 (54.86)	240.08 (55.01)	.51

*ASA,* American Society of Anesthesiologists; *BMI,* body mass index; *BUN,* blood urea nitrogen; *CHF,* congestive heart failure; *COPD,* chronic obstructive pulmonary disease; *WBC,* white blood cells.

Data are presented as number (%) or mean (standard deviation).

Four machine learning models were evaluated for their ability to predict 30-day risk of DVT after EVTA as the outcome: Classification and Regression Trees (CART), Optimal Classification Trees (OCT), Random Forests, and Extreme Gradient Boosting (XGBoost).

### Missing data imputation

Of the 34 features in the data, 17 had missing values. It has been noted that missing values in datasets are often not randomly distributed,^13^ which could lead to biased results if patients with incomplete data are removed. To avoid this, the missing values were filled in using Optimal Imputation, a method that has been shown to perform better than other state-of-the-art optimization techniques.^14^

### Training/testing data splits

The dataset was then divided into two sections—training data, which comprised 16,161 cases, and independent testing data, consisting of 5388 cases. This division follows the standard machine learning practice of a 75/25 split, where the majority of the data is allocated for training purposes, while a portion is reserved for evaluation.

### Grid-search model training

We use the training data to train potential model candidates including CART, Random Forest, OCT, and Gradient Boosted Trees with different hyperparameters. These models are selected since they are all tree-based methods that will allow more interpretability access for clinical use. We also aim to compare all these models to select the most state-of-the-art model for this particular task. Class imbalance, where one class has significantly more samples than the other, can negatively impact model performance and lead to biased results where the majority class is overrepresented. To address this, we employed sample weighting for each model, which artificially increases the weight of the minority class. This helps ensure a more balanced representation of both classes in the model. Finally, we test all possible hyperparameter combinations for each model by conducting a grid search and selecting the combination that achieved the highest area under the curve (AUC). The model was finally then evaluated using area under the receiver operator curve (AUC ROC), sensitivity, and specificity.

The parameters that were tuned include: for CART: maximum tree depth and minimum number of samples per leaf; for Random Forest: maximum tree depth, minimum number of samples per leaf, number of trees in the forest, and cost complexity parameter; for OCT: maximum tree depth and minimum number of samples per leaf; and for Gradient Boosted Trees: number of tree estimators, step size shrinkage, and minimum loss reduction required to make a further partition on a leaf node of the tree.

### Shapley additive exPlanations interpretability analysis

The selected best model was interpreted using Shapley Additive exPlanations (SHAP). SHAP is a game-theoretic approach that explains the output of a machine learning model by quantifying the individual contributions of each feature to the final prediction, both for individual samples and the overall population. This method also allows for the examination of linear and nonlinear interactions between features.^15^

### Software requirements

**Fig 1:**
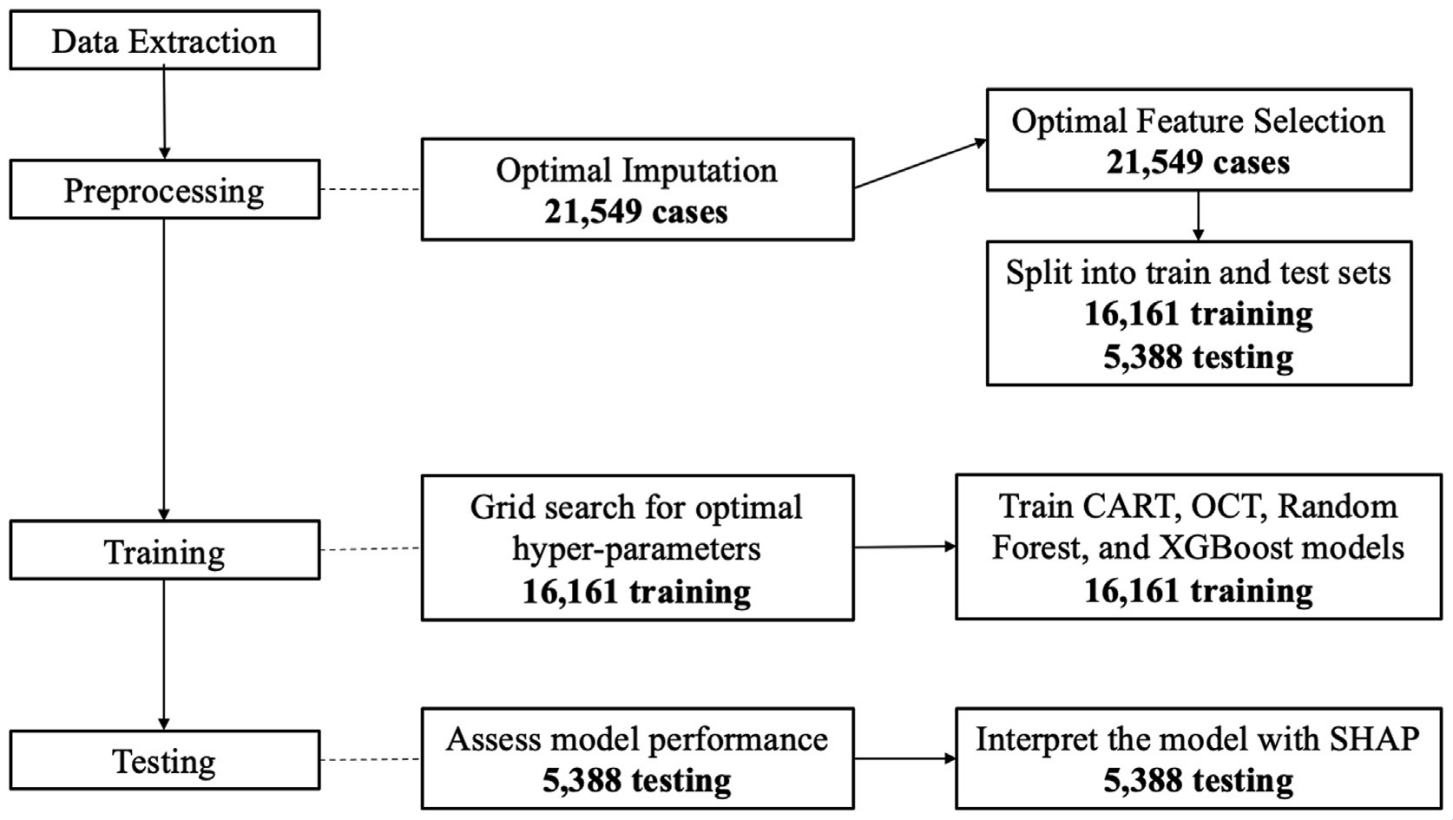
Criteria for patient inclusion. *CART*, Classification and Regression Trees; *OCT*, Optimal Classification Trees; *SHAP*, Shapley Additive exPlanations; *XGBoost*, Extreme Gradient Boosting.

These models were developed and evaluated using Python version 3.8.13 (packages: interpretableai, numpy, pandas, scikit-learn, xgboost, shap, math). The full procedure is illustrated in Fig 1. This study was approved by the Massachusetts General Brigham Institutional Review Board. The data were analyzed anonymously, and consent was waived for this study.

### Statistical analysis

Data processing and analysis were performed using Python 3.8.13. Summary statistics were presented as total counts and frequencies for categorical variables and as mean and standard deviation values for continuous variables. The differences of data distribution between data sets for both categorical and continuous variables were assessed by χ^2^ test and *t*-test, respectively.

### Ethics statement

Ethical standards were upheld throughout the study and approved by the institutional review board of Massachusetts General Hospital. A waiver of consent was obtained from the institutional review board committee due to the use of de-identified patient data and no identifiable medical risk, relying solely on pre-existing information in patient medical records.

## Results

### Cohort

Our study comprised a total of 21,549 patients. Table I provides characteristics of both the training and testing cohorts, as well as the entire study population. On average, patients included in the model development had a mean age of 54.0 years (SD, 13.8 years) and a mean body mass index (BMI) of 30.45 kg/m^2^ (SD, 7.64 kg/m^2^). The majority of individuals in the cohort were female, accounting for 67.2% of the total, whereas 22.5% were categorized as ASA class ≥3. Additionally, 12% of the cohort reported a history of smoking.

### Model performance

The top-performing machine learning model for predicting the 30-day risk of DVT following EVTA was XGBoost. This model demonstrated an AUC-ROC of 0.711, with an accuracy of 0.985, sensitivity of 1.00, and specificity of 0.047 when evaluated on the testing set. The results of all models on the training and testing sets are shown in Table II.

**Table II:** Comparison of model performance and metrics of the four models

Model	AUC in train set	AUC in test set	Accuracy	Sensitivity	Specificity
OCT	0.759	0.580	0.616	0.547	0.617
CART	0.637	0.558	0.680	0.419	0.684
Random Forest	0.789	0.670	0.767	0.442	0.773
Gradient Boosted Trees (XG Boost)	1	0.711	0.985	1.000	0.047

*AUC,* Area under the curve; *CART,* classification and regression trees; *OCT,* optimal classification trees; *XGBoost,* extreme gradient boosting.

**Fig 2:**
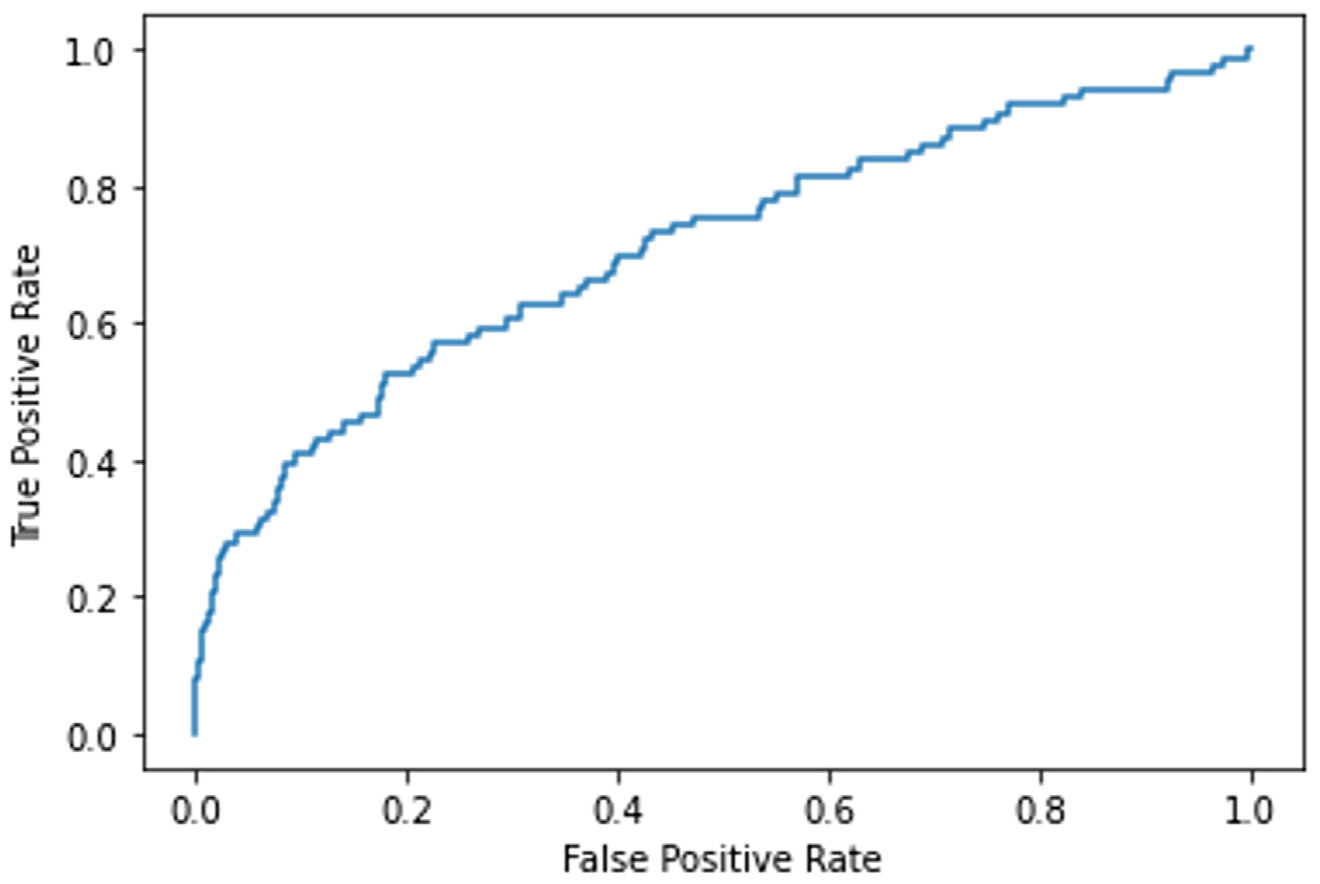
Area under the receiver operating curve (AUC-ROC) for Extreme Gradient Boosting (*XGBoost*) model.

**Fig 3:**
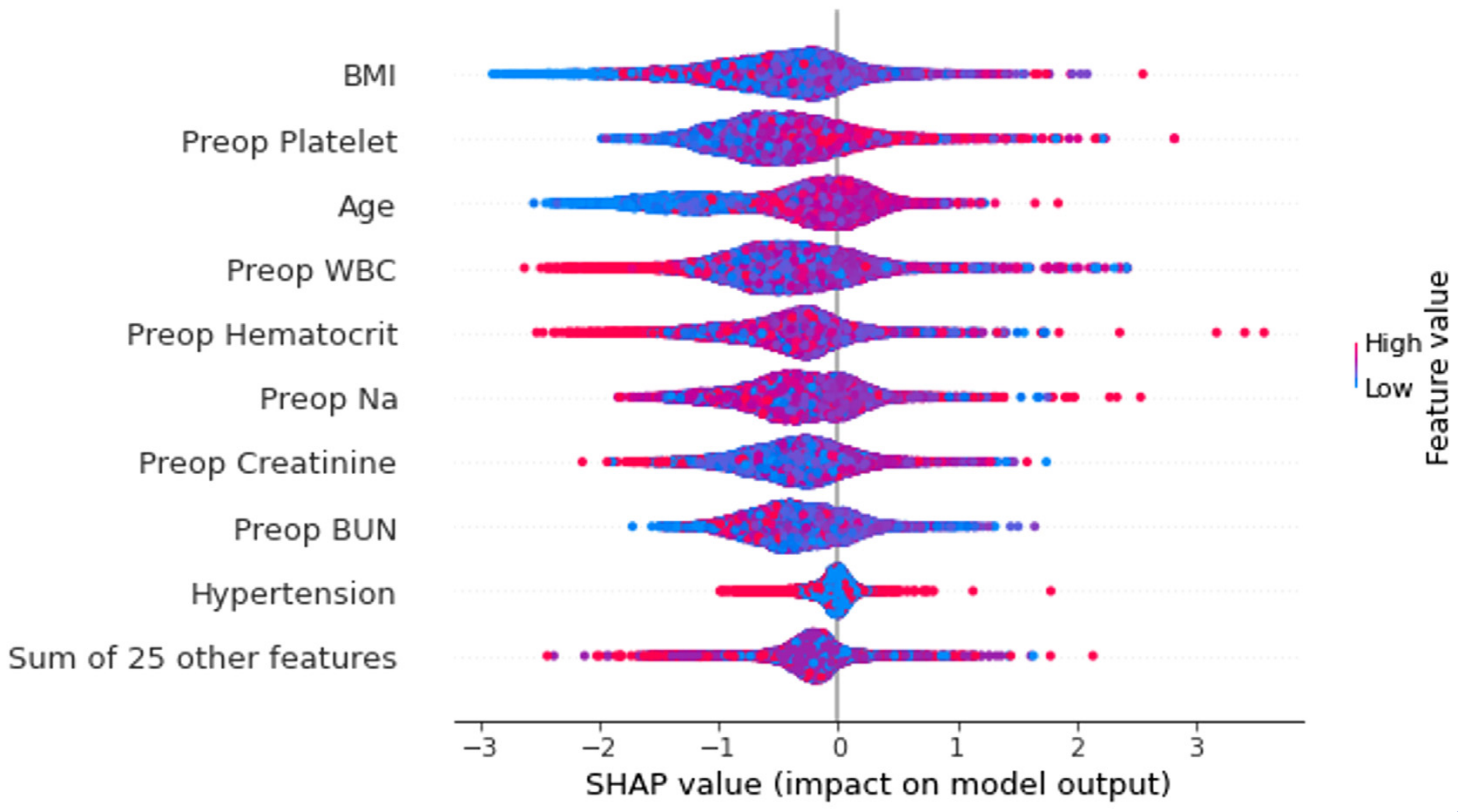
Feature importance plot for all-variable model, where the *red* indicates a higher feature value, and *blue* indicates a smaller feature value. The features are ranked by their contribution importance to the model, and thus body mass index (*BMI*) is considered the most important feature. The rest of the 25 features not demonstrated have their aggregated importance summarized as the last point. *BUN*, Blood urea nitrogen; *Na*, sodium; *SHAP*, Shapley Additive exPlanations; *WBC*, white blood cells.

Fig 2 illustrates the AUC-ROC curve. This model utilized 34 features, including demographic data, high-risk physiologic factors, and various laboratory metrics such as hematocrit, blood urea nitrogen, creatinine, white blood cell (WBC) count, platelet count, elective surgery status, renal conditions, dyspnea, and the presence of an open wound or infection (see Table I for more details). The Random Forest model, an ensemble machine learning method, integrates multiple decision trees to form a final prediction by taking into account the outputs from all trees. Its collective prediction is achieved by averaging the individual predictions, enhancing accuracy by harnessing the combined strength of several weak models. Fig 3 presents the SHAP summary plot, which identifies the key features in the model, such as BMI, preoperative platelets count, and age. Feature color indicates the direction of impact, with higher values (*red*) for preoperative platelet count linked to a greater risk of DVT, as reflected by higher SHAP values.

**Fig 4:**
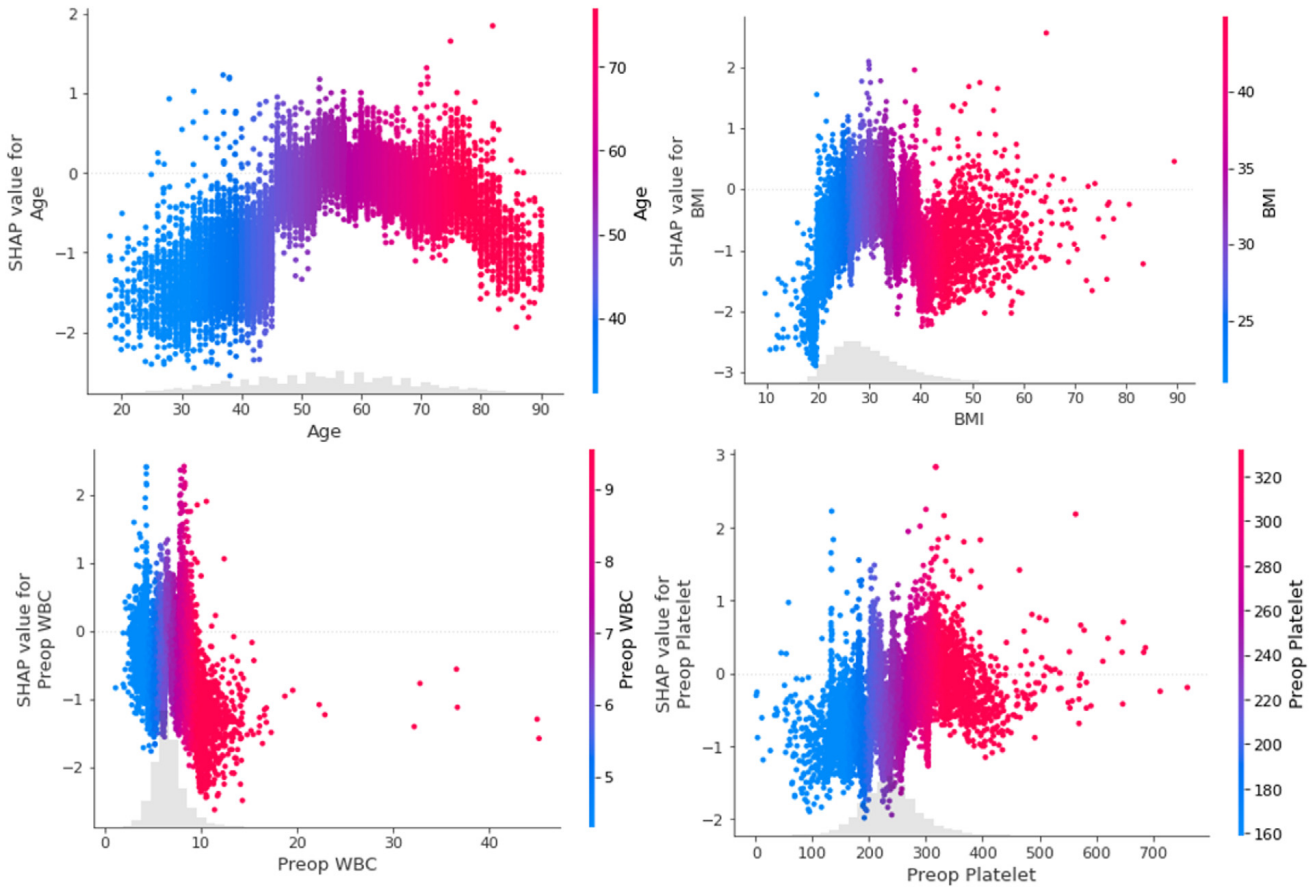
Shapley Additive exPlanations (SHAP) dependence plots of the top four importance features. *BMI*, Body mass index; *WBC*, white blood cells.

In addition, Fig 4 demonstrates the SHAP dependence plot of BMI, preoperative platelet count, age and preoperative WBC count, the top four most important features from the gradient boosted trees model vs its SHAP value. For example, for the age-dependence plot (Fig 4), as age increases above a threshold of 46 years, patients become more likely to develop DVT, although this likelihood starts decreasing again at around 60 years. Positive SHAP values indicate that this feature value contributes to the positive prediction of developing DVT, whereas negative SHAP values indicate a contribution to predicting no DVT event.

### An example of the power of personalized use of the predictive model

**Fig 5:**
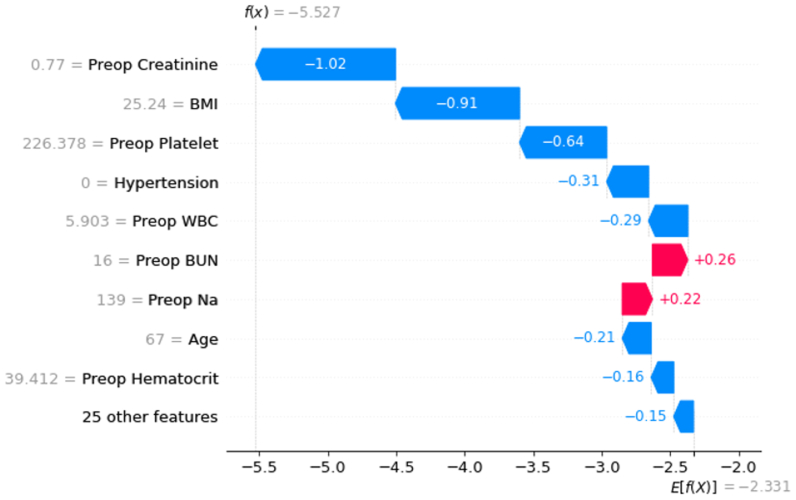
Visualization with Shapley Additive exPlanations (SHAP) plots of Extreme Gradient Boosting (*XGBoost*) model performance using all variables in an example patient. *BMI*, Body mass index; *BUN*, blood urea nitrogen; *Na*, sodium; *WBC*, white blood cells.

One of the main advantages of our proposed approach is its ability to demonstrate the effects of predictive features on each individual outcome. Using SHAP, we can see how each feature contributes to the prediction of outcome for each instance in the data. This contribution will include both the direction and the magnitude of the variable effect given the feature values for that particular sample. In this section, we highlight the significant benefit of employing the proposed personalized usage of the predictive model based on an example (Fig 5): a 67-year-old woman, self-identified as White, was admitted with an ASA class 2, had a BMI of 25 kg/m^2^, and showed no abnormal clinical variables. For this patient, the SHAP plots of XGBoost model performance, using all variables, exhibits the log of the population level probability of having DVT (E[f(X)]), and each bar represents the contribution of each feature to either increase or decrease the probability of having DVT. We observe that all features represented as most important for this patient also correlate to the population-level most important features. The feature contribution direction also corresponds well to the previous SHAP interaction plots, where having an age of 67 years pushed the probability of DVT negative by log (−0.21) and having BMI of 25.25 kg/m^2^ similarly pushed the probability by log (−0.91). Meaning when the patient has an older age and a high BMI, their risk of DVT in fact decreases.

### Difference between the two models

We observe that the order of top contributing features that are selected in the two examples of the model based on the whole population and on a single patient are different. The reasoning for this is that, although certain features aggregately are more important than others, when down to individual patients, depending on their other various characteristics, the effect of influence of those features are unique to that patient. From a technical point of view, recall that each individual follows potentially different decision paths of the decision trees among the XGBoost and Random Forest models. This means that different individuals’ final predicted outcome may depend on different features. Although certain features are consistently considered importantly aggregately across the entire population, others become more specific and dependent on individual patients. For example, patient A’s decision path only follows preoperative platelets, and thus SHAP indicates these two are most significant for this patient. However, aggregately, other patients strongly rely on BMI and preoperative platelets, as well as others, then SHAP would indicate a different ranking for the full cohort.

## Discussion

In this study, we have developed four machine learning models aimed at predicting the risk of DVT occurrence within 30 days following EVTA. The Random Forest and XGBoost models were shown to perform well (AUC, 0.67 and 0.71, respectively). We interpreted the models using SHAP to gain an understanding of the importance and direction of influence of each feature. Among patients undergoing lower extremity EVTA, the most important predictors of DVT were age, BMI, hypertension, and preoperative lab values. For each of the top four features, we split the cohort into subgroups and performed SHAP analysis to identify differences between plots. We compared the performance of several different machine learning models on the task of predicting 30-day procedure-related risk of DVT development: OCT, CART, Random Forests, and XGBoost. The CART and OCT models performed similarly on the AUC metric, but the specificity and accuracy of the CART model were higher at 0.68. The specificity of the XG Boost model was the lowest at 0.047, which indicates that this model generated a large number of false positives. Conversely, the Random Forest model achieved high specificity but low sensitivity, demonstrating an inability to perform the key task of identifying patients at risk of DVT development.

For 30-day risk prediction, the XG Boost model achieved the highest AUC at 0.711, with good sensitivity and accuracy compared with the OCT, CART, and Random Forest models, which have low sensitivity at 0.54, 0.41, and 0.44, respectively. These results indicate that XGBoost, a tree-based ensemble method, may be the best option for predicting DVT risk. This is primarily due to its ability to capture nonlinear relationships between variables found in the ACS-NSQIP database, making it more suitable than traditional linear models.

Although prognostic tools for VTE exist for patients with various underlying conditions,^16^^,^^17^ to the best of our knowledge, this study represents the first attempt to utilize an AI-based tool for predicting the risk of developing DVT following EVTA. In a prior study focusing on the early diagnosis of DVT in patients undergoing hip arthroplasty, XGBoost performed as the top-performing model, achieving an impressive AUC of 0.982.^18^

Conventional methods for identifying risk factors usually involve constructing risk models through univariate or multivariate regression techniques. However, these approaches often struggle to capture interactions and nonlinear relationships among variables.^18^ In contrast, machine learning models are better equipped to recognize more intricate patterns among variables. In medical decision-making, ensuring the interpretability of these models is essential, as transparency is a key concern when incorporating AI into clinical insights. Using the SHAP algorithms, which are based on the Shapley value concept from game theory, we calculate each feature’s contribution to the prediction, allowing for a clearer understanding of how different features influence the model’s outcomes. In this study, we leverage SHAP explanatory methods to understand both the importance of each feature across all predictions as well as the direction of influence of each feature for individual predictions following model development. The key variables we have identified align closely with established knowledge regarding the factors contributing to post-EVTA risks of DVT development, underscoring the utility of our model. For example, physiologic high-risk factors including BMI, age, preoperative WBC count, and preoperative platelet value are the top four most important predictors for DVT development. The well-documented association between DVT and factors such as age and weight further validates our model’s recognition of physiologic high-risk factors as pivotal predictors.^19^, ^20^, ^21^, ^22^

Other important predictors for DVT, preoperative platelet and WBC counts, also demonstrate the clinical relevance of the model. Studies in the 1970s using radiolabeled leukocytes have shown uptake of WBCs into venous thrombi, whereas accumulation of polymorphonuclear neutrophils on the abluminal side of the endothelium following occlusion of veins, first led to the speculation that ‘white-cell’-induced endothelial damage in contributing to venous thrombosis in humans.^23^ The exposure of the collagen-rich wall is purported to trigger platelet aggregation and subsequent leukocyte sequestration, creating a nidus for thrombus propagation.^23^ Although the role of WBCs in the natural progression of venous thrombus is multifaceted, recent findings indicate that recruited polymorphonuclear neutrophils may instigate thrombosis by generating neutrophil extracellular traps.^23^^,^^24^

Our model emphasizes the significance of multiple variables, providing interpretable insights into the 30-day risk of DVT. The XGBoost model performs well, particularly in its incorporation of diverse patient factors. However, the potential for overfitting due to data imbalance necessitates caution. The study has limitations, including reliance on the ACS-NSQIP database, which primarily represents large teaching hospitals, potentially limiting generalizability. The database only captures 30-day outcomes, precluding long-term DVT risk assessment, and lacks key clinical variables such as intervened vessels, operator expertise, and procedural variations. Additionally, procedural uniformity is assumed through Current Procedural Terminology code filtering. Performance differences between the training and testing sets suggest possible overfitting, although independent test set results remain strong. SHAP analysis reveals correlations but does not imply causation. Furthermore, the class imbalance, with only 1.59% of positive cases, may bias the model. To address this, balancing techniques were employed to improve class parity.

## Conclusions

We have developed and validated an XGBoost interpretable model that enables physicians to predict which patients with superficial venous insufficiency have higher risk of developing DVT within 30 days following EVTA. This model may help us personalize the medical decisions to minimize the risk of DVT development in these patients.

## Author contributions

Conception and design: AT, YM AG, HK DB, DD

Analysis and interpretation: AT, YM, JA, DB, DD

Data collection: YM

Writing the article: AT, YM

Critical revision of the article: AT, JA, AG, HK, DB, DD

Final approval of the article: AT, YM, JA, AG, HK, DB, DD

Statistical analysis: YM, JA

Obtained funding: Not applicable

Overall responsibility: DB, DD

AT and YM contributed equally to this article and share co-first authorship.

DB and DD contributed equally to this article and share co-senior authorship.

## Funding

None.

## Disclosures

None.

## References

[1] Nellis J.M., Obi A.T., Powell C.A., Wakefield T.W. (2022). Treatment and contemporary outcomes associated with adjunct tourniquet use during phlebectomy of complex, voluminous truncular varicosities. J Vasc Surg Venous Lymphat Disord.

[2] Whiteley M.S. (2022). Current best practice in the management of varicose veins. Clin Cosmet Investig Dermatol.

[3] Fletcher S.E., Jasuja S., Lawler L.P., Moriarty J.M. (2021). Catheter directed thrombolysis and mechanical intervention in deep venous thrombosis: what is the status after the ATTRACT Trial?. Postgrad Med.

[4] Itoga N.K., Rothenberg K.A., Deslarzes-Dubuis C., George E.L., Chandra V., Harris E.J. (2020). Incidence and risk factors for deep vein thrombosis after radiofrequency and laser ablation of the lower extremity veins. Ann Vasc Surg.

[5] Kakkos S.K., Gohel M., Baekgaard N. (2021). Editor's choice - European Society for Vascular Surgery (ESVS) 2021 clinical practice guidelines on the management of venous thrombosis. Eur J Vasc Endovasc Surg.

[6] Twine C.P., Kakkos S.K., Aboyans V. (2023). Editor's choice - European Society for Vascular Surgery (ESVS) 2023 clinical practice guidelines on antithrombotic therapy for vascular diseases. Eur J Vasc Endovasc Surg.

[7] Healy D.A., Twyford M., Moloney T., Kavanagh E.G. (2021). Systematic review on the incidence and management of endovenous heat-induced thrombosis following endovenous thermal ablation of the great saphenous vein. J Vasc Surg Venous Lymphat Disord.

[8] Healy D.A., Kimura S., Power D. (2018). A systematic review and meta-analysis of thrombotic events following endovenous thermal ablation of the great saphenous vein. Eur J Vasc Endovasc Surg.

[9] Ten Cate V., Prins M.H. (2017). Secondary prophylaxis decision-making in venous thromboembolism: interviews on clinical practice in thirteen countries. Res Pract Thromb Haemost.

[10] Skeik N., Westergard E. (2020). Recommendations for VTE prophylaxis in medically Ill patients. Ann Vasc Dis.

[11] Shohat N., Ludwick L., Sherman M.B., Fillingham Y., Parvizi J. (2023). Using machine learning to predict venous thromboembolism and major bleeding events following total joint arthroplasty. Sci Rep.

[12] Qin L., Liang Z., Xie J. (2023). Development and validation of machine learning models for postoperative venous thromboembolism prediction in colorectal cancer inpatients: a retrospective study. J Gastrointest Oncol.

[13] Hamilton B.H., Ko C.Y., Richards K., Hall B.L. (2010). Missing data in the American College of Surgeons national surgical quality improvement program are not missing at random: implications and potential impact on quality assessments. J Am Coll Surg.

[14] Bertsimas D., Pawlowski C., Zhuo Y.D. (2018). From predictive methods to missing data imputation: an optimi- zation approach. J Mach Learn Res.

[15] Lundberg S.M., Lee S.I. (2017). Advances in Neural Information Processing Systems [Internet].

[16] Deng R.X., Zhu X.L., Zhang A.B. (2023). Machine learning algorithm as a prognostic tool for venous thromboembolism in allogeneic transplant patients. Transpl Cell Ther.

[17] Fresard M.E., Erices R., Bravo M.L. (2020). Multi-objective optimization for personalized prediction of venous thromboembolism in ovarian cancer patients. IEEE J Biomed Health Inform.

[18] Ding R., Ding Y., Zheng D. (2023). Machine learning-based screening of risk factors and prediction of deep vein thrombosis and pulmonary embolism after hip arthroplasty. Clin Appl Thromb Hemost.

[19] Wang T., Yang S., Wang Z., Guo J., Hou Z. (2023). Incidence and risk factors of admission deep venous thrombosis in nonagenarians and centenarians with intertrochanteric fracture: a retrospective study. J Orthop Surg Res.

[20] Engbers M.J., van Hylckama V.A., Rosendaal F.R. (2010). Venous thrombosis in the elderly: incidence, risk factors and risk groups. J Thromb Haemost.

[21] Yang G., De Staercke C., Hooper W.C. (2012). The effects of obesity on venous thromboembolism: a review. Open J Prev Med.

[22] Klovaite J., Benn M., Nordestgaard B.G. (2015). Obesity as a causal risk factor for deep venous thrombosis: a Mendelian randomization study. J Intern Med.

[23] Saha P., Humphries J., Modarai B. (2011). Leukocytes and the natural history of deep vein thrombosis: current concepts and future directions. Arterioscler Thromb Vasc Biol.

[24] Sheils C.R., Dahlke A.R., Kreutzer L., Bilimoria K.Y., Yang A.D. (2016). Evaluation of hospitals participating in the American College of Surgeons national surgical quality improvement program. Surgery.

